# Expression of IL-1β, IL-6, TNF-α, and a-MMP-8 in sites with healthy conditions and with periodontal and peri-implant diseases: A case-control study

**DOI:** 10.34172/joddd.40958

**Published:** 2024-06-24

**Authors:** Renzo Guarnieri, Rodolfo Reda, Dario Di Nardo, Gabriele Miccoli, Francesco Pagnoni, Alessio Zanza, Luca Testarelli

**Affiliations:** ^1^Private Practice, Treviso, Italy; ^2^Department of Oral and Maxillofacial Sciences, Sapienza University of Rome, Rome, Italy; ^3^Department of Prosthodontics and Implantology, Saveetha Dental College and Hospitals, Saveetha Institute of Medical and Technical Sciences, Chennai, India; ^4^Operative Research Unit of Dentistry, Policlinico Universitario Campus Bio-Medico Foundation, Via Alvaro del Portillo, Roma, Italy

**Keywords:** a-MMP-8, GCF, Gingival crevicular fluid, Interleukins, Peri-implant crevicular fluid, PICF

## Abstract

**Background.:**

This study evaluated the gingival crevicular fluid (GCF) and Peri- implant crevicular fluid (PICF) concentrations of interleukin-1 beta (IL-1β), interleukin-6 (IL-6), tumor necrosis factor-alpha (TNF-α) and active metalloproteinase-8 (a-MMP-8) in sites with healthy conditions vs. sites affected by periodontitis (PER) and peri-implantitis (PIM).

**Methods.:**

Periodontally healthy (PH) sites with PER, sites with peri-implant health (PIH), and sites with PIM were investigated intra-individually, according to the inclusion criteria of each group. Probing pocket depth (PPD), plaque index, gingival index, and the presence or absence of bleeding on probing (BoP) were evaluated. In GCF and PICF samples, IL-1β, IL-6, and TNF-α were quantified by ELISA Duoset® kit in combination with Ultramark® micro-ELISA digital reader; a-MMP8 concentration was analyzed by a chairside test (Perio/ImplantSafe®) in combination with a digital reader (ORALyzer®).

**Results.:**

The concentrations of IL-6 and IL-1β, TNF-α, and a-MMP-8 were significantly higher in the PIM and PER sites compared to healthy sites (*P*<0.05). Significantly higher concentrations of IL-1β and a-MMP-8 were found in PIM vs. PER sites (*P*<0.05), while the concentrations of IL-6 and TNF-α did not differ between the PIM and PER groups (*P*>0.05).

**Conclusion.:**

aMMP-8, IL-6, IL-1β, and TNF-α presented higher GCF/PICF concentrations in diseased periodontal and peri-implant sites. However, only the concentrations of IL-1β and a-MMP-8 were significantly higher in PIM than in PER sites.

## Introduction

 Periodontitis (PER) and peri-implantitis (PIM) are polymicrobial inflammatory diseases that destroy the tissues supporting the tooth/implant.^1^ Although the etiology of PER and PIM is currently recognized to be similar,^2^ a different pathogenesis, mediated by a different immune-inflammatory host response to the microbial insult, seems to be responsible for the tissue damage.^3,4^ In contrast to PER, PIM lesions show a differing cell profile with high numbers of B cells, osteoclasts, and neutrophils.^5^ PER and PIM also show a different quantitative transcript profile, suggesting the prevalence of bacterial response in PER tissues, with the prevalence of innate immune response in PIM tissues.^6^ Moreover, the destruction of tissues in PIM appears to be of significantly greater severity than occurs in PER.^7,8^

 In healthy conditions, the periodontal and peri-implant tissues that are in close contact with the dental biofilm show a low-grade active immune response, which is physiological.^9^ The transition to a pathological condition begins with an immune-cellular activation process, which results in dysregulation in the production of inflammatory mediators such as cytokines, chemokines, prostaglandins, and proteolytic enzymes that, in turn, alter the connective tissue and bone metabolism.^9-11^ During the last years, an increasing interest in the assessment of some inflammatory cytokines, such as interleukin-1 beta (IL-1β), interleukin-6 (IL-6), tumor necrosis factor-alpha (TNF-α), and enzymes, such as matrix metalloproteinase-8 (MMP-8) within the gingival crevicular fluid (GCF) and peri-implant crevicular fluid (PICF) has been targeted for periodontal and peri-implant disease detection and prediction to elucidate a broad overview of the pathogenesis of these diseases.^12-16^ IL-1β, IL-6, and TNF-α are released from the cells of the gingival epithelium, dendritic cells, connective tissue fibroblasts, macrophages, and neutrophils. They mediate the production of prostaglandin E2, leukotrienes, and platelet-activating factor and promote osteoclast activation and bone resorption. IL-1β regulates the degradation of extracellular matrix components of the plasminogen system and the collagenase activity in inflammation and wound healing. TNF-α induces fibroblast apoptosis and reduces the repair capacity of the peri-implant tissue. Furthermore, IL-1β, IL-6, and TNF-α induce tissue destruction and bone resorption by activating collagenase and through the receptor activator of nuclear factor-kappa B ligand, which stimulates osteoclast differentiation.^12-16^ Enzymes, such as MMP-8, are produced by neutrophils, fibroblasts, and osteoclasts, resulting in collagen degradation of connective tissue and alveolar bone.^17^ Although several studies on GCF/PICF IL-1β, IL-6, TNF-α, and MMP-8 levels have been performed for periodontal and peri-implant disease detection, so far, limited research has compared the levels of these biomarkers in clinical situations of periodontal/peri-implant health (PIH) and disease.^18-20^ Since the disease susceptibility, the immune response, and the inflammatory mediator’s profile, being individual, vary between subjects,^21^ the present study evaluated the intra-individualGCF/PICF concentrations of IL-1β, IL-6, TNF-α, and a-MMP-8 in sites with healthy conditions vs. sites affected by PER and PIM. The null hypothesis of this study was that there are differences in GCF/PICF IL-1β, IL-6, TNF-α, and MMP-8 levels between periodontal and peri-implant diseases. In addition, this study sought to assess the differences between healthy and diseased sites.

## Methods

 A clinical and radiographic investigation of dental implant therapy was performed in 2021 after approval by the Ethics Committee of the Campus Bio-Medico University of Rome. Adult subjects were selected ( > 18 years old), male or female, with generalized PER, periodontal health, PIM, or PIH. All eligible individuals were invited to participate in the study and were thoroughly informed of its nature, potential risks, and benefits. The study was conducted in accordance with the Declaration of Helsinki, and participants agreeing to participate in the study signed an informed consent form. For inclusion in the study, patients had to be > 18, systemically healthy, partially edentulous, with one or more missing teeth restored with fixed implant-supported restorations loaded for at least 12 months. Subjects with systemic diseases affecting the healing process (e.g., uncontrolled diabetes mellitus) were excluded from this study. Smoking patients, patients with liver/kidney or salivary gland dysfunction, under cancer therapy or organ transplant, pregnant or lactating women, individuals using antibiotics or immunosuppressive medication within the last three months, those needing antibiotics for infective endocarditis prophylaxis during dental procedures, those having orthodontic appliances, patients presenting oral mucosal inflammatory conditions, HIV-positive patients, and those with a history of hepatitis were excluded. Furthermore, patients whose implants had mobility or needed guided bone regeneration or sinus elevation before implant placement were also excluded. Participants selected (n = 140) had been treated with dental implants at the same office between 2010 and 2020. Thirty patients declined the invitation, bringing the total number of included patients to 112 (80%). In addition, the mean implant installation time was five years (range: 5–10 years).

 Each site categorized with PER, after conventional periodontal treatment of scaling and root planing, had to present probing pocket depth (PPD) ≥ 4 mm, bleeding on probing (BoP), and radiographic marginal bone loss ≥ 3 mm (Stage III to IV). Each site categorized as a healthy peri-implant site (the PIH group) had to present the absence of peri-implant signs of soft tissue inflammation (redness, swelling, and BoP) and the absence of further additional bone loss following initial healing. Each site categorized with PIM had to present BoP and/or suppuration, with a probing depth (PD) > 4 mm, and radiographic bone loss > 3 mm, with at least 50% of peri-implant bone remaining (otherwise, the implant was considered lost). Baseline bone level measurements on radiographs from implant surgery were reduced by 1 mm to compensate for the anticipated initial bone remodeling. If the individual had more than one implant affected by PIM and more than one tooth affected by PER only, one implant and one tooth were evaluated.

 The PD (6 sites per tooth), plaque index, gingival index, and modified sulcular bleeding index were employed to assess the periodontal clinical status. The clinical status of peri-implant tissues was evaluated by assessing the PD (six sites per implant) and corresponding indices for implants, including a modified plaque index (mPI),^21^ simplified gingival index (sGI),^22^ and a modified bleeding on probing index (mBOPI).^21^ To avoid the risk of IL-1β, IL-6, TNF-α, and aMMP-8 fluctuation due to mechanical irritation, the clinical examination was performed a week before PICF and GCF sampling.

 The sampling site was prepared for GCF/PICF analysis by removing excess saliva with a short, gentle blast of air. Using tweezers, a sterile PICF collection strip was placed apically as deeply as possible into the sulcus at the sampling site. Following the manufacturer’s instructions, the a-MMP-8 levels were determined by the a-MMP-8 PoC/chairside mouthrinse test (PerioSafe®) in combination with a digital reader (ORALyzer®), whereas IL-1β, IL-6, and TNF-α were quantified by ELISA-Duoset® kit in combination with Ultramark® micro-ELISA digital reader.

 A computer-assisted measurement automatically provided by a software program (VixWin Platinum, Gendex) was used for radiographic measurements of digital radiographs taken with the same radiologic device. The distance between the prosthetic connector and the peri-implant alveolar bone crest was measured to obtain vertical bone loss. These measurements were made by a single examiner who was previously trained and were used to complement the diagnosis of PIM.

###  Examiner calibration

 Calibration exercises for clinical parameters and IL-1β, IL-6, TNF-α, and aMMP-8 estimation were performed in ten sites before the actual study. Clinical recordings were done twice by a single examination within one month. The order of patients was masked and changed between the examinations. The examiner received training before the study regarding the use of each index employed for periodontal and peri-implant examinations. PD was measured using a pressure-sensitive probe (Florida Probe, Gainesville, FL, USA), and the estimation was judged to be reproducible if the agreement within ± 1 mm between repeated measurements was at least 90%. The intra-examiner agreement between the two measurements was found to be 91%. A different examiner, who was blinded to the clinical records of the patients, carried out the fluid sampling.

###  Statistical analysis

 IL-1β, IL-6, TNF-α, and aMMP-8 were considered the primary outcome. Based on the IL-1β, IL-6, TNF-α, and a-MMP-8 differences between the groups, the sample size was determined according to previously published data. The following parameters were used for the sample size calculation: a minimum expected difference between means of 0.5, standard deviations on the difference between means of 0.7, an effect size of 0.71, a beta error of 10%, and one-tailed alpha error of 5%, with an 80% power. This resulted in a required sample size of 56 patients. However, based on the anticipated individual variations in IL-1β, IL-6, TNF-α, and a-MMP-8 responses and the specific study design accounting for potential losses and refusals, the sample size was doubled. These calculations thus estimated a minimum of 112 patients. Data were analyzed using IBM SPSS Statistics (Version 23.0 for Windows; IBM Corp). The Wilcoxon signed-rank test was used for intra-individual pairwise comparisons of IL-1β, IL-6, TNF-α, and aMMP-8 levels and PPD/BoP; Spearman’s rank correlation test was used to determine intra-individual correlations between sites regarding IL-1β, IL-6, TNF-α, and aMMP-8 levels. For all parameters, *P* values < 0.05 were considered statistically significant.

## Results

 A total of 295 GCF/PICF samples (112 in periodontally healthy [PH] sites, 57 in PER sites, 94 in PIH sites, and 31 in PIM sites) collected from 112 patients were examined. Table 1 presents the mean periodontal clinical parameters ( ± SD) of the study population.

**Table 1:** Mean periodontal clinical parameters ( ± SD) of the study population

**Site**	**PI/mPI**	**GI/mGI**	**PD (mm)**	**% BoP**
PH (n = 112)	0.84 ± 0.73	1.08 ± 0.18	2.2 ± 0.3	14.7 ± 5.5
PER (n = 57)	1.48 ± 0.84^(^*^)^	1.5 ± 0.33	3.74 ± 0.7^(^**^)^	72.9 ± 16.1^(^***^)^
PIH (n = 94)	1.05 ± 0.13	1.04 ± 0.14	2.6 ± 0.8	12.1 ± 5.1
PI (n = 32)	1.25 ± 0.39	1.37 ± 0.41^(^†^)^	5.5 ± 1.2^(^††^^)^	82.3 ± 17.3^(^†††^^^)^

PH, periodontally healthy; PER, periodontitis; PIH, peri-implant health; PI, Peri-implantitis. Wilcoxon signed-rank test: significant difference from PH, ^(^*^)^*P* = 0.024; ^(^**^)^*P* = 0.037; ^(^***^)^*P* = 0.028 Significant difference from PIH, ^(^†^)^*P* = 0.041, ^(^††^)^*P* = 0.014, ^(^†††^)^*P* = 0.033,^(^^^)^ significant difference from PER, ^(^^^)^*P* = 0.028, ^(^^^^)^*P* = 0.011.

 The mean age was 59.8 ± 12.3 years, and the majority (64) were female. Seventy-seven subjects (60%) had a diagnosis of PER and 22 (20%) PIM. The mean number of dental implants was 2.7 (range: 1–5), and the mean number of residual teeth was 22.4 (range: 12–32). The mean number of sites with PPD ≥ 4 was 8.4 (range: 4–42).

 The mean PI/mPI, PD, and percentage of BoP values were significantly (*P* < 0.017, *P* < 0.032, and *P* < 0.042, respectively) higher in PER/PIM sites than in healthy sites. Compared to PER, PIM sites showed significantly higher values of PD and BoP (*P* < 0.035 and *P* < 0.042, respectively). Table 2 presents the GCF/PICF levels of all biomarkers studied.

**Table 2:** The means of IL-6, IL-1β, TNF-α, and aMMP-8 levels in GCF/PICF samples and intra-individual pairwise comparisons between groups

	**PH**	**PER**	**PIH**	**PI**
IL-1β (pg/mL)	24.12 ± 7.2	41.19 ± 15.78 (*)	22.73 ± 3.1	59.32 ± 10.04 (†) (^)
IL-6 (pg/mL)	4.22 ± 3.4	7.71 ± 56.1 (**)	4.38 ± 4.3	8.63 ± 5.8 (††)
TNF-α (pg/mL)	14.14 ± 1.67	51.08 ± 12.04 (***)	15.19 ± 2.93	47.88 ± 11.39 (†††)
aMMP-8 (ng/mL)	11.58 ± 3.1	17.51 ± 9.3 (****)	12.42 ± 2.9	29.8 ± 10.6 (††††) (^^)

PH, periodontally healthy; PER, periodontitis; PIH, peri-implant health; PI, Peri-implantitis. Wilcoxon signed-rank test: significant difference from PH (*) *P* = 0.043, (**) *P* = 0.022; (***) *P* = 0.031, (****) *P* = 0.027; significant difference from PIH (†) *P* = 0.036, (††) *P* = 0.028, (†††) *P* = 0.039, (††††) *P* = 0.055; significant difference from PER (^) *P* = 0.043, (^^) *P* = 0.022.

**Figure 1:**
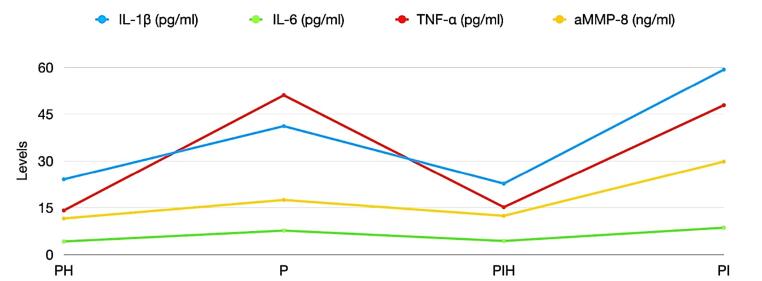
Means of the intra-individual levels of the investigated biomarkers. PH, periodontally healthy; PER, periodontitis; PIH, peri-implant health; PI, Peri-implantitis

 The concentration of a-MMP-8, IL-6, IL-1β, and TNF-α were significantly higher in the PIM and PER sites compared to the healthy sites (Figure 1). Significantly higher concentrations of IL-1β and a-MMP-8 were found in PIM vs. PER sites, while the concentrations of IL-6 and TNF-α did not differ between the PIM and PER groups.

## Discussion

 Several studies have attempted to clarify the role of cytokines in periodontal and peri-implant tissues’ immuno-inflammatory response; however, the literature presents scarce comparative data regarding the concentration of these biomarkers in PER and PIM and their role in the progression of these diseases.^9,18,19,23^ The present study evaluated differences in CGF/PICF levels of IL-1β, IL-6, TNF-α, and aMMP-8 in healthy, PER, and PIM sites. The null hypothesis was to verify differences in levels of these biomarkers between periodontal and peri-implant diseases. The results indicated that, compared to the healthy sites, the concentration of IL-6, IL-1β, and TNF-α were significantly higher in both PIM and PER sites. These results agree with previously published data indicating that the levels of these proinflammatory cytokines in GCF and PICF were higher than in healthy controls and that their secretion is closely related to the progression of diseases.^12-15,18,19^ In addition, current studies indicated that only the concentration of IL-1β was significantly higher in PIM sites compared to PER sites, while the concentrations of IL-6 and TNF-α did not differ between the PIM and PER groups.

 Although some evidence suggests that IL-1β, IL-6, and TNF-α act synergistically to initiate and propagate inflammation and stimulate osteoclasts, inducing bone resorption,^24,25^ recent reports indicated that they have different roles in the two types of immune response, acquired and innate. IL-6 is prevalently involved in adaptive immunity and immune reactions to infection.^26^ Many studies have shown that IL-6 is related to infectious diseases, promoting the differentiation of B cells, the secretion of antibody proteins, and the activation of cytotoxic T cells.^27-29^ Involvement of IL-6 in the pathogenesis of periodontal disease is well-recognized, and IL-6 has been shown to possess potential characteristics to be used as a valid, sensitive, and specific biomarker for periodontal diseases.^30^ Literature data on IL-6 expression in PIM are contrasting, and no conclusive evidence has been found to prove their usefulness as markers of peri-implant disease.^31-36^ Based on the current study, indicating that the concentrations of IL-6 did not differ between the PIM and PER groups, it is possible to hypothesize that PER and PIM present the same degree of adaptive immune reaction to infection.

 IL-1β is produced essentially by macrophages, followed by neutrophilic granulocytes, monocytes, lymphocytes, and fibroblasts, and possesses a broad spectrum of inflammatory and immunologic properties playing a crucial role in the innate immune system.^26^ Levels of IL-1β failed to discriminate between periodontal health and disease.^37^ On the contrary, several investigations reported that the levels of IL-1β were positively correlated to failing dental implants at the patient and site level, indicating IL-1β as a promising candidate in differentiating PER from healthy implants.^36,38,39^ Two distinct signals are needed for the expression, activation, and release of active IL-1β, namely primary and secondary signals.^40^ Several bacterial products, among which lipopolysaccharide, work as a primary stimulus, resulting in the up-regulation of the expression of pro-IL-1β and accumulation of pro-IL-1β intracellularly in macrophages. A secondary signal induced by direct interactions of various bacterial species or bacterial components (such as for *Salmonella typhimurium* or *Francisella tularensis*) or cell-surface interactions (such as for *Listeria monocytogenes*, *Staphylococcus aureus*, *Aggregatibacter actinomycetemcomitans* or *Porphyromonas gingivalis*), through the activation of the inflammasome complex and caspase-1 activation is ultimately required for IL-1β secretion.^41-48^ Recent studies indicate that titanium wear ions/particles may also function as a secondary stimulus to activate the inflammasome in macrophages, resulting in the release of active IL-1β from cells.^49^ Titanium particles from dental implant surfaces have been observed in soft and hard peri-implant tissues. Although their impact on the pathogenesis of PIM and in the host osteoimmunoinflammatory response is still indefinite, some studies have related the presence of these particles to peri-implant tissue inflammatory processes.^48-52^ In vitro investigations indicated that mice immune cells stimulated by lipopolysaccharides in the presence of titanium ions showed increased release of IL-1β was involved in bone resorption.^53^ In addition, titanium particles have been shown to increase the release of IL-1β by macrophages and induce secretion of receptor activator of nuclear factor-kappa B ligand upon entering T-cells, stimulating bone resorption.^54^ The long-standing accumulation of oral microbial biofilm on implant surfaces combined with mechanical strain may cause titanium implant surfaces to deteriorate,^55-57^ inducing a pathogenic cycle from the interaction between titanium and bacterial plaque that leads to definitive alterations on the titanium surface. In contrast, bacterial attachment and growth occur on the corroded surface. The pathogenic cycle may lead to definitive alterations on the titanium surface, whereas bacterial attachment and growth occur on the corroded surface. According to the results of the current study, it can be assumed that the higher IL-1β levels found in PIM sites, compared to PER sites, might depend on the presence of ions/particles released from dental implants, which contribute to the inflammatory reactions by stimulating inflammasome activation and IL-1β secretion in macrophages, which was substantially enhanced in the presence of microbial stimuli. However, further studies with histochemical and immunohistochemical analyses are needed to confirm this hypothesis.

 In the present study, compared to healthy tissues, the levels of TNF-α showed an increase in both diseased periodontal and peri-implant tissues. However, no significant differences in levels of this mediator have been found between PER and PIM sites. In a previous study by Jansson et al,^20^ the intra-individual TNF-α profile has been reported to not differ between sites diagnosed with PER and those diagnosed with PIM but differ between healthy tooth and healthy implant sites. Since little data is present in the literature, further investigations are needed to comprehend the possible differential role of TNF-α in inflammation and the progression of periodontal and peri-implant diseases.

 Regarding aMMP-8 levels, the present study showed an increase in this enzyme in PIM, compared to PER, suggesting its higher involvement in the pathogenesis of PIM. MMP-8, also known as “neutrophil collagenase,” is synthesized during the myelocyte stage of the development of the neutrophils, stored in the specific or secondary granules and released first upon activation of the cells by reactive oxygen species, tissue and plasma proteinases, or opportunistic microbial proteinases (alone or in concert).^58^ Once activated, the catalytically competent MMP, such as active MMP-8 (aMMP-8), acts as a potential initiator of interstitial collagenolysis at inflammatory sites. In an experimental study comparing ligature-induced PIM and PER lesions in mice, Hiyari et al^59^ histologically assessed soft tissue changes, including the destruction of the collagen matrix via MMP-8. Ligature-treated implants showed increased immunoreactivity of MMP-8 as compared to teeth. Authors attributed this observation to the fact that implants may present with some natural basal level of inflammation, making implants more readily prone to developing an inflammatory reaction than natural teeth.^60,61^ Different involvement of the metalloproteinases in the pathogenesis of PER and PIM has also been suggested by Borsani et al,^62^ who characterized the distribution of metalloproteinases both in the gingival epithelium and stroma in periodontal and peri-implant soft tissues. Another study indicated that fibroblasts from PIM granulation tissue showed the up-regulation of mRNA collagenase and reduced gene expression for tissue inhibitors of matrix metalloproteinases compared to cells collected from chronic PER granulation tissue.^63^ Therefore, it cannot be excluded that the production of collagenase may augment the destructive events in local tissue and, by a vicious circle, contribute to the further release of particles, which may partly be responsible for tissue destruction.

 The evaluation of the biomarkers used in the present study allowed us to elucidate possible differences between the pathogeneses of PER and PIM. However, the results must be interpreted cautiously due to some limitations. Firstly, this study evaluated a relatively low number of subjects and biomarkers in each group. Secondly, most of the study population consisted of PER-prone individuals, which may limit the generalizability of the results to a general population. Furthermore, due to the cyclic progression of periodontal and peri-implant diseases, the biomarkers of immune-inflammatory events responsible for tissue breakdown may not always be detected with a single moment of fluid collection.

## Conclusion

 Within the limitations of the current study, the results indicated that the GCF/PICF levels of IL-6, IL-1β, TNF-α, and aMMP-8 differed between healthy teeth and sites diagnosed with PER and between healthy implant sites and sites diagnosed with PIM. In addition, they indicated differences in IL-1β and aMMP-8 concentrations between sites affected by PER and PIM, suggesting different pathogenic mechanisms in these diseases.

## Competing Interests

 The authors declare no conflicts of interest.

## Ethical Approval

 The study was conducted in accordance with the Declaration of Helsinki and approved by the Ethics Committee of Università Campus Bio-Medico di Roma (protocol code Prot. PAR 30.21 (OSS) ComEt CBM-30/03/2021).

## Funding

 This research received no external funding.
